# Robust taxonomic classification of uncharted microbial sequences and bins with CAT and BAT

**DOI:** 10.1186/s13059-019-1817-x

**Published:** 2019-10-22

**Authors:** F. A. Bastiaan von Meijenfeldt, Ksenia Arkhipova, Diego D. Cambuy, Felipe H. Coutinho, Bas E. Dutilh

**Affiliations:** 1 Theoretical Biology and Bioinformatics, Science for Life, Utrecht University, Utrecht, The Netherlands; 2 Centre for Molecular and Biomolecular Informatics, Radboud University Medical Centre, Nijmegen, The Netherlands; 3 Instituto de Biologia, Universidade Federal do Rio de Janeiro, Rio de Janeiro, RJ Brazil; 4 Present Address: Evolutionary Genomics Group, Departamento de Produccíon Vegetal y Microbiología, Universidad Miguel Hernández, Campus San Juan, San Juan, 03550 Alicante, Spain

## Abstract

Current-day metagenomics analyses increasingly involve de novo taxonomic classification of long DNA sequences and metagenome-assembled genomes. Here, we show that the conventional best-hit approach often leads to classifications that are too specific, especially when the sequences represent novel deep lineages. We present a classification method that integrates multiple signals to classify sequences (Contig Annotation Tool, CAT) and metagenome-assembled genomes (Bin Annotation Tool, BAT). Classifications are automatically made at low taxonomic ranks if closely related organisms are present in the reference database and at higher ranks otherwise. The result is a high classification precision even for sequences from considerably unknown organisms.

## Background

Metagenomics, the direct sequencing of DNA from microbial communities in natural environments, has revolutionized the field of microbiology by unearthing a vast microbial sequence space in our biosphere, much of which remains unexplored [1–3]. With increases in DNA sequencing throughput, metagenomics has moved from analysis of individual reads to sequence assembly, where increases in sequencing depth have enabled de novo assembly of high-quality contiguous sequences (contigs), sometimes many kilobases in length [4]. In addition, current state-of-the-art encompasses binning of these contigs into high-quality draft genomes, or metagenome-assembled genomes (MAGs) [5–8]. The advance from short reads to contigs and MAGs allows the metagenomics field to answer its classical questions [9], “who is there?” and “what are they doing?” in a unified manner: “who is doing what?”, as both function and taxonomy can be confidently linked to the same genomic entity. Because assembly and binning can be done de novo, these questions can be applied to organisms that have never been seen before, and the discovery of entirely novel phyla is common still [8].

Several efficient tools for taxonomic classification of short-read sequences have been developed over the years, reflecting the read-based focus of the time. Most tools consider each read as an independent observation, whose taxonomic origin can be estimated by identifying best-hit matches in a reference database, either on read, K-mer, or translated protein level (see [10] for an overview). Widely used programs such as Kraken [11] (K-mer based), CLARK [12] (discriminative K-mer based), and Kaiju [13] (protein-based) can process hundreds of thousands of sequencing reads per second. Without compromising accuracy, still faster approaches use mixture modeling of K-mer profiles, as implemented in FOCUS [14]. Sometimes a Last Common Ancestor (LCA) algorithm is applied to allow for multiple hits with similar scores as the best hit (e.g., Kraken, MEGAN [15]).

Similar approaches are often applied to contigs, with classification often based on the best hit to a reference database. Although fast, the best-hit approach can lead to spurious specificity in classifications, for example when a genomic region is highly conserved or recently acquired via horizontal gene transfer (HGT) from a distantly related organism. As we will show below, the problem is particularly grave when the query contigs are very divergent from the sequences in the database, i.e., they are distantly related to known organisms. Whereas specificity (correctly classified/total classified) can be increased when only classifications at higher taxonomic ranks are considered, this approach is not desirable as taxonomic resolution is unnecessarily lost for query contigs that are closely related to known organisms.

Depending on their length, contigs may contain multiple open reading frames (ORFs), each of which contains a taxonomic signal. Integrating these signals should enable a more robust classification of the entire contig, yet surprisingly few tools exist that integrate distributed signals for contig classification. The viral-specific pipeline MetaVir2 [16] assesses the classification of up to five ORFs encoded on a contig. Recently, the MEGAN long-read algorithm was introduced [17], which allows users to taxonomically classify long sequences such as those generated by Oxford Nanopore Technologies or Pacific Biosciences sequencers. The algorithm works by partitioning the sequence into intervals based on the location of hits of a LAST [18] search.

In contrast, for taxonomic classification of MAGs, it is common to include information from multiple ORFs. Since the classification of complete genomes by using phylogenetic trees of multiple marker genes is well-established [19], MAG classification has followed these best practices. Some steps in the process can be automated, including initial placement in a low-resolution backbone tree by CheckM [20], specific marker gene identification, and backbone tree taxon selection by phyloSkeleton [21], and many tools are available for protein alignment, trimming, tree building, and display. However, interpretation of the resulting phylogeny remains a critical manual step, making this approach for genomic taxonomy a laborious task that does not scale well with the increasing number of MAGs being generated (see, e.g., [7]).

Here we present Contig Annotation Tool (CAT) and Bin Annotation Tool (BAT), two taxonomic classifiers whose underlying ORF-based algorithm is specifically designed to provide robust taxonomic classification of long sequences and MAGs. Both tools exploit commonly used tools for ORF calling and homology searches. They require minimal user input and can be applied in an automated manner, yet all aspects are flexible and can be tuned to user preferences.

### Benchmarking classification of sequences from novel taxa

Taxonomic classifiers are often benchmarked by testing them on sequences from novel taxa, i.e., that are not (yet) in the reference database (e.g., as in the CAMI challenge [22], and [11, 12, 14]). Alternatively, unknown query sequences can be simulated by using a “leave-one-out” approach, where the genome that is being queried is removed from the database (e.g., [13, 17]). However, due to taxonomic biases in database composition, other strains from the same species, or other species from the same genus, may still be present. Thus, the leave-one-out approach does not reflect the level of sequence unknownness that is often encountered in real metagenomes, where the query sequences may be only distantly related to the ones in the reference database. A benchmark better suited to address this novelty is a “leave-entire-taxa-out” approach also known as clade exclusion, where all related sequences belonging to a certain taxonomic rank are removed from the database (e.g., [11, 23, 24]).

Here, we rigorously assess the performance of taxonomic classification tools by developing an extensive database reduction approach at different taxonomic ranks, where novel species, genera, and families are simulated by removing all the sequences of entire taxa from the database. In a second benchmark, we classified the high-complexity CAMI dataset [22]. We show that the algorithm of CAT and BAT allows for the correct classification of organisms from known and unknown taxa and outperforms existing methods, especially for sequences that are highly unknown (i.e., with no close relatives in the database). Third, we used BAT in a real-world challenge to classify a large, recently published set of 913 MAGs from the cow rumen [7] that represent a wide range of novelty at all taxonomic ranks, and whose published taxonomic classifications involved extensive phylogenetic analyses.

## Results and discussion

To test the performance of our newly developed taxonomic classification tools CAT and BAT, we thoroughly tested them in three independent benchmarks: (1) A clade exclusion experiment with increasing levels of sequence unknownness, (2) the high-complexity gold standard CAMI assembly, and (3) a recently published set of MAGs where the BAT classifications are compared to the published taxonomic classifications.

### Contig classification with CAT

#### Benchmark 1: Classification of increasingly unknown sequences

**Fig. 1:**
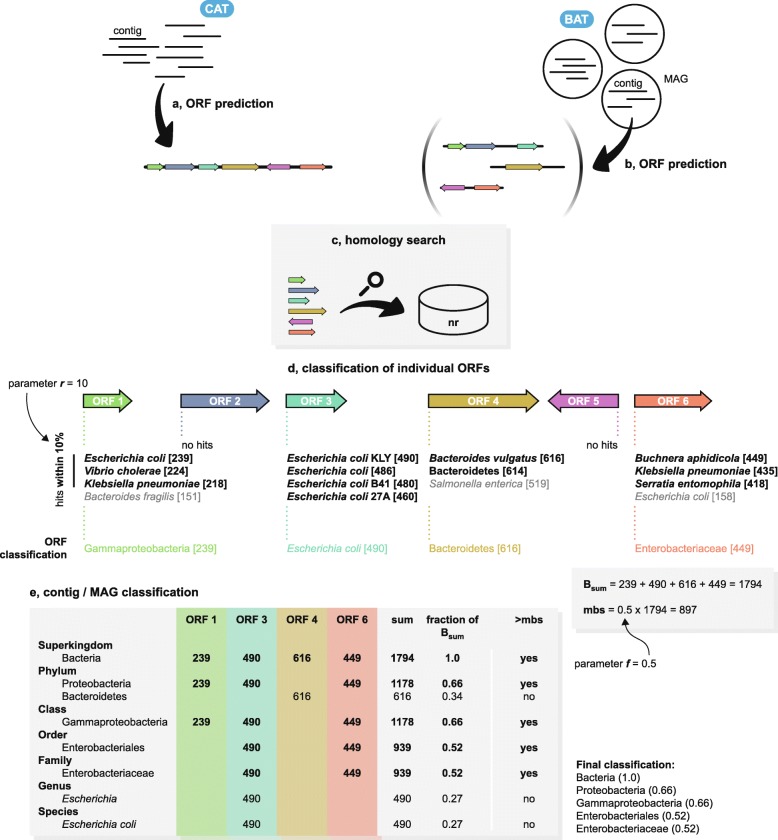
Contig and MAG classification with CAT and BAT. **a**, **b** Step 1: ORF prediction with Prodigal. CAT analyses all ORFs on a contig, BAT analyses all ORFs in a MAG. **c** Step 2: predicted ORFs are queries with DIAMOND to the NCBI non-redundant protein database (nr). **d** Step 3: ORFs are individually classified based on the LCA of all hits falling within a certain range of the top hit (parameter *r*), and the top-hit bit-score is assigned to the classification. Bit-scores of hits are depicted within brackets. Hits in gray are not included in final annotation of the ORF. Parameter *f* defines minimal bit-score support (mbs). **e** Step 4: contig or MAG classification is based on a voting approach of all classified ORFs, by summing all bit-scores from ORFs supporting a certain classification. The contig or MAG is classified as the lowest classification reaching mbs. The example illustrates the benefit of including multiple ORFs when classifying contigs or MAGs; a best-hit approach might have selected *Bacteroides vulgatus* or Bacteroidetes if an LCA algorithm was applied as its classification, as this part has the highest score to proteins in the database in a local alignment-based homology search. In the example, only six taxonomic ranks are shown for brevity; in reality, CAT and BAT will interpret the entire taxonomic lineage

**Fig. 2:**
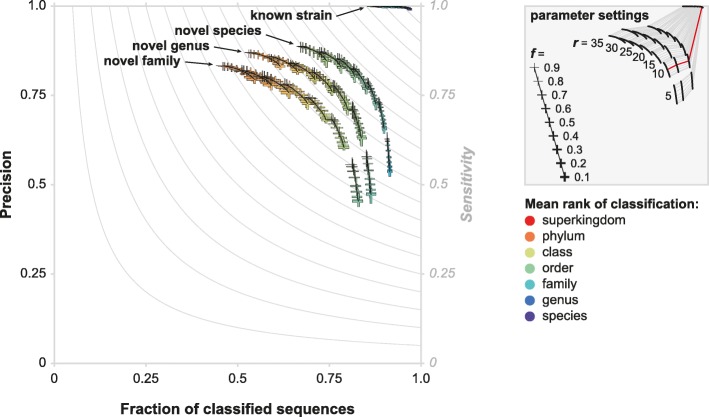
Classification performance of CAT for different levels of unknownness across a range of parameter settings. Thickness of markers indicates values of the *f* parameter; runs with similar *r* parameter values are connected with black lines. Markers indicate maximum and minimum values out of ten benchmarking datasets, bars cross at the means. Color coding indicates the mean taxonomic rank of classification averaged across the then benchmarking datasets (minimum and maximum values not shown for brevity). Gray lines in the plot depict sensitivity, which is defined as the fraction of classified sequences times precision. Runs with equal parameter settings are connected in the parameter settings figure, showing that CAT achieves a high precision regardless of unknownness of the query sequence, by classifying sequences that are more unknown at higher taxonomic ranks. Default parameter combination (*r* = 10, *f* = 0.5) is shown in red

We used CAT (Fig. 1) to classify ten simulated contig sets in the context of four reference databases with different levels of simulated unknownness, representing query sequences from (A) known strains, (B) novel species, (C) novel genera, and (D) novel families (see the “Methods” section). To assess the effect of the two key user parameters, *r* (hits included within *range* of top hits) and *f* (minimum *fraction* classification support), on precision, fraction of classified sequences, sensitivity, and taxonomic rank of classification, we ran CAT with a wide range of possible parameter values against all four reference databases (Fig. 2). This parameter sweep revealed a trade-off between the classification precision on the one hand and the taxonomic resolution and the fraction of classified sequences on the other hand. This general trend can be understood by considering that classifications at a low taxonomic rank (i.e., close to the species rank, high taxonomic resolution) will inevitably be increasingly imprecise, especially if closely related organisms are absent from the reference database. This might be resolved by classifying sequences at a higher taxonomic rank, but this leads to increased numbers of sequences not being classified or classified at trivially informative taxonomic ranks such as “cellular organisms” or “root.”

The *r* parameter, which governs the divergence of included hits for each ORF, has the largest effect. As increasing *r* includes homologs from increasingly divergent taxonomic groups, their LCA is pushed back and classifications at low taxonomic ranks are lost, resulting in fewer classified sequences and classifications at lower taxonomic resolution (i.e., at higher taxonomic ranks), but with higher precision. The *f* parameter, which governs the minimum bit-score support required for classifying a sequence, has a smaller effect. Decreasing *f* results in classifications that are based on evidence from fewer ORFs, leading to more tentative classifications at lower taxonomic ranks. As a result, more sequences are classified at lower taxonomic ranks, albeit with a lower precision.

As a user increases *r* and *f*, this will increasingly result in high-rank classifications that are correct but ultimately uninformative. When low values of *r* and *f* are chosen, the classifications will be more specific (i.e., at a lower taxonomic rank) but more speculative (i.e., precision goes down). Based on the parameter sweep described above, we set the default values for CAT contig classification to *r* = 10 and *f* = 0.5 (red line in the legend of Fig. 2). Note that this value of *f* = 0.5 results in at most one classification, since > 50% of the bit-score supports that classification.

#### Comparison to state-of-the-art taxonomic classifiers

We compared classification by CAT in this first benchmark to (1) the recently published LAST+MEGAN-LR algorithm [17], (2) the widely used Kaiju algorithm [13], and (3) a conventional best-hit approach with DIAMOND [25]. Kaiju, designed for short-read classification, uses a best-hit approach with an LCA algorithm if equally good top-hits are found. Its underlying algorithm allows for the classification of long sequences as well and has recently been used as such [17, 26, 27]. Final Kaiju classification is based on the hit with the maximum exact match (MEM), or on the highest scoring match allowing for mismatches (Greedy).

**Fig. 3:**
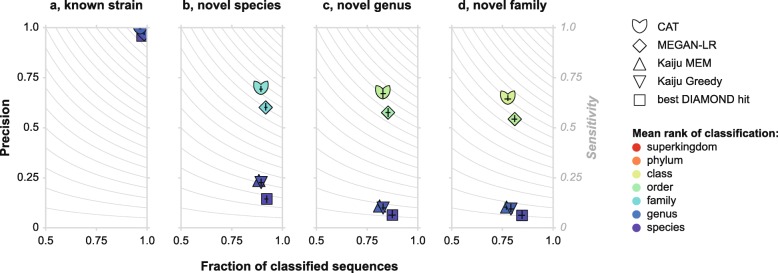
Classification performance of CAT, LAST+MEGAN-LR, Kaiju, and DIAMOND best-hit for different levels of unknownness. **a** Classification of known sequences. **b**–**d** Classification of simulated novel taxa for different levels of divergence from reference databases. Black bars indicate maximum and minimum values out of ten benchmarking datasets, bars cross at the means. Color coding indicates the mean taxonomic rank of classification averaged across the then benchmarking datasets (minimum and maximum values not shown for brevity)

When classifying simulated contigs against the full reference database (known strains), all programs showed a similar precision and fraction of classified sequences (Fig. 3a). The mean taxonomic rank of classification is slightly higher for CAT and LAST+MEGAN-LR than for the other approaches (Additional file 1: Table S1), reflecting the conservative LCA-based classification strategies of the former two. DIAMOND best-hit does not use an LCA algorithm, and Kaiju only in cases where multiple hits have identical scores, and thus, they classify contigs according to the taxonomic rank of their match in the reference database.

When novel species, genera, and families were simulated by removing related sequences from the database, precision declined rapidly for DIAMOND best-hit and Kaiju (Fig. 3b–d). The classifications called by these approaches are often too specific, because in databases where closely related sequences are absent, the singular best hit may still match a sequence that is annotated at a low taxonomic rank, although this annotation cannot match that of the query. This spurious specificity can be seen in the mean rank of classification, which stays close to the species rank, even when sequences from the same species, genus, or family were removed from the database (Fig. 3b–d, Additional file 1: Table S1). CAT and LAST+MEGAN-LR clearly perform better in the face of such uncharted sequences. With default parameter settings, CAT has higher precision and sensitivity than MEGAN-LR and classifications are made at slightly higher taxonomic ranks.

Precision for CAT and LAST+MEGAN-LR increases when the sequence contains more ORFs with a DIAMOND hit to the database, whereas this is not the case for DIAMOND best-hit and Kaiju (Additional file 2: Figure S1). Algorithms that integrate multiple taxonomic signals are thus well suited for taxonomic classification of long metagenomic sequences and MAGs (see below), but even the majority of contigs in our benchmarking sets that contained a single ORF are still classified correctly (Additional file 2: Figure S1).

#### Sequences are classified correctly and automatically at the appropriate taxonomic rank

As a solution to the spurious specificity of the best-hit approach described above, classifications are sometimes assigned to a higher taxonomic rank such as genus, family, or even phylum. However, applying a rank cutoff may unnecessarily sacrifice taxonomic resolution in cases where the query sequences do have close relatives in the reference database and classification at a low taxonomic rank would be justified. Additional file 2: Figure S2 shows that application of a rank cutoff to the best-hit classifications (e.g., reporting all classifications at the genus or phylum rank) does not solve the problem of spurious specificity as effectively as CAT does. CAT classifications have a higher precision than a best-hit cutoff on a rank comparable to its mean rank. For example, when novel families are simulated, the mean rank of classification for CAT is between order and class, and precision is much higher than best-hit classifications on those ranks, with a similar fraction of classified sequences (Additional file 2: Figure S2d). Importantly, CAT has the highest precision on a per rank basis of any of the tested tools (Additional file 2: Figure S3, Additional file 1: Table S2). This shows that CAT approach of integrating multiple taxonomic signals across a sequence leads to better classifications.

As shown in Fig. 2, the ORF-based voting algorithm ensures a high precision regardless of the level of unknownness of the query sequences, i.e., whether closely related sequences are present in the reference database or not. In some circumstances, taxonomic resolution is traded for precision: when classifying sequences that are more distantly related to the sequences in the reference database, hits will have weaker bit-scores and match sequences that are taxonomically more diverse. As a result of these conflicting signals, the algorithm automatically increases the taxonomic rank when classifying more divergent query sequences. Thus, no rank cutoff is needed for precise classifications, regardless of the composition of the metagenome.

#### Benchmark 2: Comparison to CAMI tools

*O*ur second benchmark consisted of classifying the high-complexity gold standard assembly of the CAMI challenge [22]. Classifying the CAMI dataset has two benefits. First, it allows us to compare CAT to any of the taxonomic classifiers tested in the CAMI challenge (referred to as “taxonomic binners” in [22]). Second, CAMI simulated novel organisms, making it a complementary benchmarking approach as compared to the database reduction method in our first benchmark.

Since novel sequences are simulated, it is crucial that search databases are used that do not contain the simulated sequences. For this reason, an “old” copy of RefSeq (dated January 30th, 2015) was supplied during the CAMI challenge. Here, we also ran CAT with that old RefSeq reference database for a fair comparison against the other tools. However, one of the advantages of CAT and BAT is that they can be run with very large protein databases and hence have a larger search space for taxonomic classification beyond RefSeq. Thus, we also ran CAT with the nr databases from a similar date (January 23, 2015) as a reference. The nr database is the default option for CAT and BAT runs.

CAT performance measures on the high-complexity gold standard contig set (Additional file 1: Table S3) are plotted in Additional file 2: Figure S4 and can be compared to Supplementary Figure 18 and Supplementary Figure 19 in [22]. Average precision increases sharply if 99% of the data are considered (i.e., removal of taxa summing up to less than 1% of the total assembly length) as opposed to 100%. This is also true for most of the tools tested in the CAMI challenge. The reason for this observation is that precision in the CAMI challenge is measured on a “per bin” basis, and erroneous classifications of single contigs thus weigh very heavily in this benchmark. If classifications that are seen in only a single or few contigs (i.e., are supported by short sequence length overall) are excluded, CAT showed very high average precision at all taxonomic ranks down to the genus level (Additional file 2: Figure S4). Accuracy and average recall were high for higher ranks and decreased towards the species level. Misclassification was very low, with misclassification rates of up to 11% only at the lowest taxonomic ranks. Notably, CAT results with nr as a reference database (Additional file 2: Figure S4b) were better than with RefSeq as reference (Additional file 2: Figure S4a) for any of the measures. Average precision stayed above 90% down to the genus level if nr was used as a reference, higher than what is achieved by any of the tools tested in the CAMI challenge (see below). This highlights the benefit of using a large reference database for taxonomic classification.

We compared CAT to the other tools tested in the CAMI challenge by downloading their performance measures from the CAMI GitHub (Additional file 2: Figure S5). The CAMI tools fall within two categories: One set of tools (taxator-tk 1.4pre1e, taxator-tk 1.3.0e, PhyloPythiaS+ mg c400, MEGAN 6.4.9) had low misclassification but also low average recall and accuracy. The other set (PhyloPythiaS+ c400, Kraken 0.10.6-unreleased, Kraken 0.10.5) had high recall and accuracy, but very high misclassification rates towards species level. In contrast, CAT managed a medium (when using RefSeq as reference database) to high (when using nr as reference database) average recall and accuracy, with a very low misclassification rate. The misclassification rate was lower than that of the CAMI tools, with the exception of taxator-tk (both versions), which classified very few sequences in general. CAT scored among the highest average precision with 99% of the data. Thus, CAT has a high average precision and combines the high average recall and accuracy of the second set of tools with the low misclassification of the first.

#### The ORF-based algorithm is fast and has a very low memory requirement

**Fig. 4:**
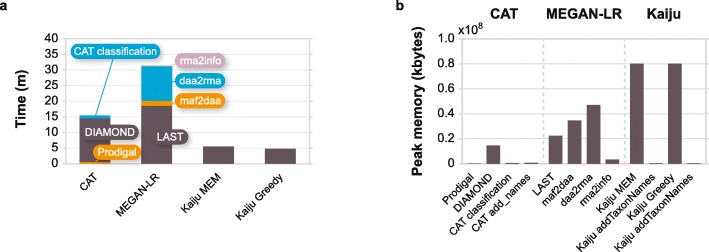
Computer resource usage by CAT, LAST+MEGAN-LR, and Kaiju. **a** Run-time and **b** peak memory usage. In **a**, classification by CAT and Kaiju includes adding taxonomic names to the classification; in **b**, these steps are depicted separately

CAT is about two times faster than LAST+MEGAN-LR (Fig. 4a) and outperforms all other programs tested in our first benchmark in terms of memory usage (Fig. 4b). The slowest and most memory intensive step is the DIAMOND search for homologs in the vast nr database, which due to the flexible nature of our implementation can be optimized for a specific use case (see Additional file 1: Table S4) or replaced by any protein aligner of a user’s choice, as can the search database.

We classified the CAMI high complexity dataset with recent versions of the tools tested in our first and second benchmarks. This analysis showed that CAT is faster than MEGAN6, LAST+MEGAN-LR, and taxator-tk and has a memory footprint that is similar to or lower than any of the tested tools (Additional file 1: Table S4).

### MAG classification with BAT

#### Benchmark 3: Classification of 913 metagenome-assembled genome bins (MAGs)

Next, we set out to apply the algorithm to MAGs, i.e., draft genomes that can be generated from metagenomes by assembly and binning. Since the typical pipeline to generate MAGs is reference database independent, they can be distantly related to known organisms. As benchmark set, we picked 913 recently published MAGs from the cow rumen [7] that represented a wide range of novelty at different taxonomic ranks (Additional file 2: Figure S6a). The published classifications were based on the placement of the MAGs in a backbone tree and subsequent refinement, a slow process that includes various manual steps and visual screening [7]. At the time of our study, the MAGs were not yet included in the reference database, providing an ideal test case for our automated classification tool BAT.

**Fig. 5:**
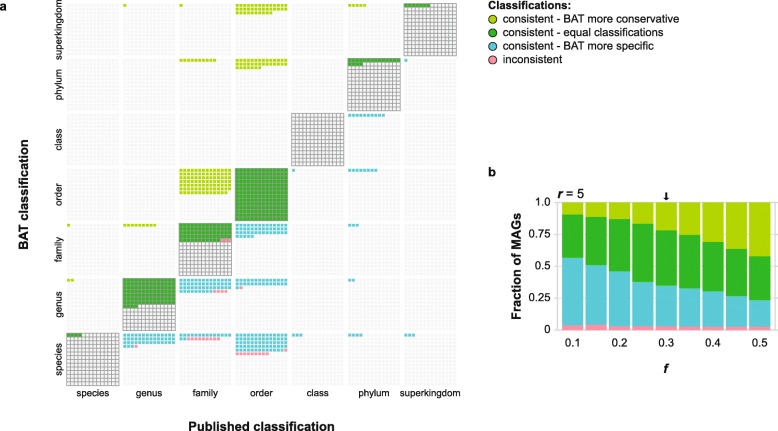
Classification of 913 MAGs with BAT. **a** Consistency between BAT classifications and published classifications with default parameter settings (*r* = 5, *f* = 0.3). **b** The mean rank of classification can be increased by increasing *f*. Arrow indicates BAT results for its default parameter settings

The 913 MAGs were previously assessed to be ≥ 80% complete and have ≤ 10% contamination and contain between 541 and 5378 ORFs each (Additional file 2: Figure S6b). We ran BAT with default parameter settings for MAGs classification (*r* = 5, *f* = 0.3). The low *r* value ensures that individual ORFs are annotated to an LCA with a relatively low taxonomic rank, as hits within 5% of the highest bit-score are considered. The low *f* value reports taxonomic classifications that are supported by at least 30% of the bit-score evidence. While this could be considered a speculative call when contigs with relatively few encoded ORFs are annotated, the much higher number of ORFs in MAGs means that even classifications with relatively low *f* values are backed by a high number of ORFs and precision is thus expected to be high (Additional file 2: Figure S1). We scored the consistency between BAT and the published classifications (Fig. 5a), dividing consistent classifications into three groups: (i) BAT can be more conservative than the published classification, i.e., BAT classifies the MAG to an ancestor of the published classification; (ii) classifications can be equal; and (iii) BAT can be more specific. Alternatively, BAT can classify a MAG inconsistently, i.e., in a different taxonomic lineage than the original publication. As shown in Fig. 5a, 885 of 913 MAGs (97%) were classified consistently with the original publication. If parameter *f* is relaxed, mean rank of classification for the MAGs increases (Fig. 5b). Importantly, decreasing the value of *f* has little effect on inconsistency rate. Thus, changing this parameter will mainly lead to a change in the rank of classification, while the taxonomic lineage will remain unchanged. Finally, classifying these MAGs with two MAG classification tools that are still under development, lastTaxa (https://gitlab.com/jfroula/lasttaxa) and GTDB-Tk (https://github.com/Ecogenomics/GTDBTk), yielded very similar results (Additional file 1: Table S5).

**Fig. 6:**
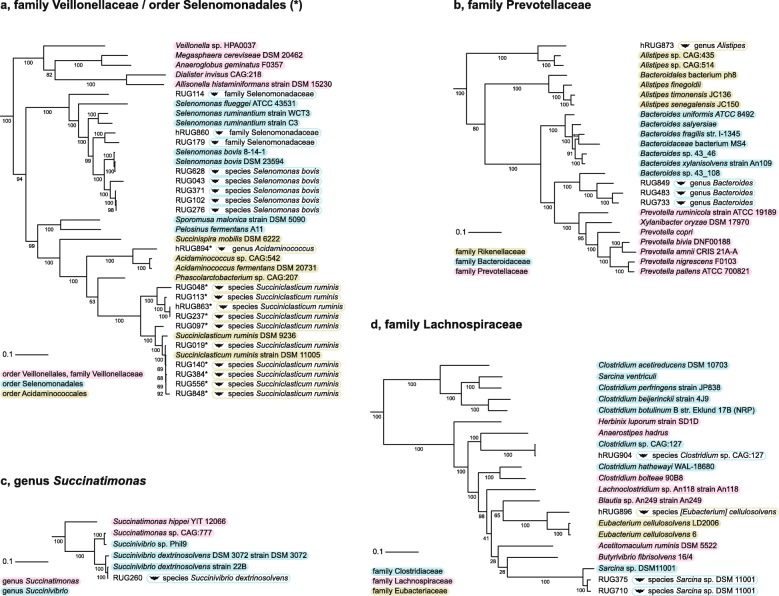
Tree placement of the 28 inconsistently classified MAGs that were assigned to five different taxa according to the original classifications (**a**–**d**). Headers of subfigures refer to the published classifications. In **a**, MAGs published as Selenomonadales are marked with an asterisk. Taxonomic classification of reference genomes is indicated in shades boxes. BAT classifications of MAGs are indicated in open boxes

To assess the taxonomy of the 28 inconsistently classified MAGs (at *r* = 5, *f* = 0.3), we placed them in a phylogenomic tree with closely related genomes and observed their closest relatives, the published classifications, and the BAT classifications. As shown in Fig. 6, BAT classified all 28 inconsistently classified MAGs more precisely and at a higher taxonomic resolution than the published classifications. Note that this may be due to these closely related reference genomes being new additions to the database since the research was performed. Together, these results highlight the benefit of using BAT for the rapid, automated, and high-resolution taxonomic classification of novel microbial lineages at a range of unknownness.

## Conclusions

Metagenomics continues to reveal novel microorganisms in all environments in the biosphere, whose genome sequences can be reconstructed with high accuracy by using high-throughput DNA sequencing and modern sequence assembly and binning tools. Taxonomically classifying these uncharted sequences remains challenging, partly because the vast natural biodiversity remains highly underrepresented in even the largest reference databases, partly because existing classifiers are built to classify short sequencing reads, and partly because interpreting trees is manual work.

We presented CAT and BAT, a set of tools that exploits DIAMOND homology searches of individual ORFs called by Prodigal, LCA annotation, and a user-definable weighting to classify long contigs and metagenome-assembled genomes (MAGs). As we have shown, these query sequences contain a wealth of information that allows their accurate taxonomic classification at appropriate taxonomic ranks, i.e., at a low rank when closely related organisms are present in the database, and at a high rank when the sequences are divergent or highly novel. We have shown that the low precision of conventional best-hit approaches when classifying novel taxa can be overcome by a voting algorithm based on classifications of multiple ORFs. Elegantly, sequences from organisms that are distantly related to those in the reference database are automatically classified at a higher taxonomic rank than known strains. ORFs on divergent sequences will hit a wider variety of different taxa both on the individual ORF level and between ORFs. Such conflict of classifications is automatically resolved by the algorithm by providing a more conservative classification, so no taxonomic cutoff rank for classification needs to be pre-defined. In metagenomes containing both known and unknown sequences, the algorithm vastly outperforms best-hit approaches and a range of state-of-the-art taxonomic classifiers in precision.

CAT and BAT supplement a modern metagenomics workflow in various ways. For example, CAT can be used after metagenome assembly to confidently classify all contigs. Since contigs are longer sequences and thus contain more information than individual reads, we expect that classification of the original reads in terms of classified contigs results in better profiling estimates than those based on the reads alone. Indeed, a comparison in [22] between taxonomic binners and dedicated taxonomic profilers (whose output is an abundance profile but not classification of individual sequences) showed that on average binners estimated taxon abundance more accurately than profilers. With increases in contig lengths due to advances in assembly algorithms and more deeply sequenced metagenomes, as well as increasingly available long-read metagenomic sequencing datasets, CAT classifications will become even more precise in the future. Moreover, BAT will rapidly provide taxonomic classifications of MAGs without requiring a full phylogenomics pipeline and subsequently visual inspection of the tree. CAT classifications of individual contigs within MAGs can be used to identify taxonomic outliers, and flag those as possible contamination. As most binning tools do not incorporate taxonomic signals (e.g., [28, 29]), CAT classification can be considered as independent evidence and might be used to decide on the inclusion of specific contigs in a MAG.

BAT provides a robust and rapid classification of MAGs in a single operation, but is not a replacement for high-confidence phylogenomic tree construction based on marker gene superalignments which remains the gold standard [19]. However, BAT queries the full NCBI non-redundant reference database (nr) and the taxonomic context is thus much bigger than any phylogenomic tree that depends on completely sequenced genomes. For example, the backbone tree of CheckM currently includes only 5656 genomes [20]. BAT classification is fully automated and can be run on a set of MAGs with minimal user input, allowing MAG classification to be scaled up considerably as we showed here for over 900 MAGs that were classified consistently with the original publication in almost all cases. Notably, in all inconsistent cases, we identified genomes that were more closely related to the BAT classification than to the published (manual) classification.

As long as sequence space is incompletely explored and reference databases represent a biased view of the tree of life [1, 3], algorithms designed to address the abundant uncharted microbial sequences will be needed to make sense of the microbial world. Decreasing sequencing costs and improvement of alignment and binning algorithms have moved metagenomics from the analysis of short reads towards contigs and MAGs, improving our understanding of microbial ecosystems to a genomic resolution. As these data will only increase in the coming years, we presented a robust solution to their specific challenges that we expect will play an important role in future metagenomics workflows.

## Methods

### Explanation of the algorithm

Both CAT and BAT take high-quality long DNA sequences in FASTA format as input (Fig. 1), such as assembled contigs or corrected long Oxford Nanopore Technologies or Pacific Biosciences reads [30, 31]. First, ORFs are predicted with Prodigal [32] in metagenome mode, using default parameter settings (genetic code 11) (Fig. 1a, b). Predicted proteins can also be independently supplied to CAT/BAT in case a user prefers a different gene caller than Prodigal.

Next, protein translations of the predicted ORFs are queried against the National Center for Biotechnology Information (NCBI) non-redundant protein database (nr) [33] using DIAMOND [25] blastp (*e* value cutoff of 0.001, BLOSUM62 alignment matrix, reporting alignments within 50% range of top hit bit-score) (Fig. 1c). The nr database is currently the largest sequence database where all sequences are assigned to clades in NCBI Taxonomy [34]. A separate BLAST tabular output file can also be supplied together with the predicted protein file, in which case CAT/BAT starts directly with classification.

Taxonomic classification of the query sequences is then carried out based on a voting approach that considers all ORFs on a query with hits to the reference database. Here, the main difference between CAT and BAT is that CAT considers ORFs on a single contig, whereas BAT considers ORFs on all contigs belonging to a MAG. CAT and BAT also have slightly different default parameter values (see below).

First, the algorithm infers the taxonomic affiliation of individual ORFs based on the top DIAMOND hits (Fig. 1d). To account for similarly high-scoring hits in potentially different clades, hits within a user-defined range of the top hit bit-score to that ORF are considered and the ORF is assigned to the LCA of their lineages (parameter *r* for *range*, by default hits with bit-scores within 10% or 5% range of the top hit bit-score are included, *r* = 10 for CAT and *r* = 5 for BAT, respectively). By adjusting parameter *r*, the user can tune how conservative CAT is in the classification of individual ORFs. For example, increasing *r* results in more divergent hits being included that together are likely to have a deeper LCA, thus leading to a more conservative ORF classification at a higher taxonomic rank. In contrast, decreasing *r* leads to a more specific classification since fewer and more similar hits will be included, likely with a narrower taxonomic range. This accounts for conserved or HGT-prone genes that are highly similar in diverse taxa by assigning them a high-rank classification. The top hit bit-score for each ORF is registered for the subsequent voting process (Fig. 1d).

Next, the query contig or MAG is evaluated by summing the bit-scores for each taxon identified among the classifications of all ORFs, as well as their ancestral lineages up to the taxonomy root (Fig. 1e). The query contig or MAG is then assigned to a taxon, if the total bit-score evidence for that taxon exceeds a cutoff value (*mbs*, minimal bit-score support), which is calculated as a fraction (parameter *f* for *fraction*) of the sum of the bit-scores of all ORFs (mbs = *f* × *B*_sum_, by default *f* = 0.5 for CAT and *f* = 0.3 for BAT). For example, if parameter *f* is set to 0.5, this means that a contig is assigned to a taxon if the majority of the sum of the bit-scores of all ORFs supports that classification (mbs = 0.5 × *B*_sum_). This is done at multiple taxonomic ranks including phylum, class, order, family, genus, and species. The algorithm stops at the taxonomic rank where the total bit-score supporting the classification drops below the minimal bit-score support value, so CAT/BAT automatically finds the lowest rank taxonomic classification that is still reliable (Fig. 1e). Note that with CAT default values (*f* = 0.5), only one classification is given per sequence, and there can be no conflicting classifications at different ranks (e.g., a species-level classification conflicting with a genus-level classification). When *f* < 0.5 is set by the user, multiple lineages at a given taxonomic rank may exceed the threshold, and all will be written to the output file. A user can decide on the appropriate (rank of) classification based on support values that represent the fraction of summed bit-score that supports the classification. While these support values are indicative of the prediction precision (Additional file 2: Figure S7a), in contrast to the total bit-score alone (Additional file 2: Figure S7b), it should be noted that they cannot be interpreted as statistical probabilities.

### Output files

For each query contig or MAG, the full taxonomic lineage of the lowest-rank supported classification is written to the output file, together with support values per rank (i.e., the fraction of *B*_sum_ that is represented by the taxon). In addition, the number of ORFs found on the contig or MAG and the number of ORFs on which the classification is based are written to the output file. An extra output file containing information about individual ORFs is also generated, including classifications of ORFs and an explanation for any ORF that is not classified. We advise the user caution when interpreting the classifications of short contigs that are based on relatively few ORFs as they will be less robust than the classifications of long contigs or MAGs (Additional file 2: Figure S1).

### Helper programs

The CAT/BAT package comes bundled with three helper utilities, “prepare,” “add_names,” and “summarise.” “Prepare” only needs to be run once. It downloads all the needed files including NCBI taxonomy files and the nr database. It constructs a DIAMOND database from nr and generates the files needed for subsequent CAT and BAT runs. Because the first protein accession in nr not always represents the LCA of all protein accessions in the entry, “prepare” corrects for this in the protein accession to taxonomy id mapping file (prot.accession2taxid). After running CAT/BAT, “add_names” will add taxonomic names to the output files, either of the full lineage or of official taxonomic ranks alone (superkingdom, phylum, class, order, family, genus, species). “Summarise” generates summary statistics based on a named classification file. For contig classification, it reports the total length of the contigs that are classified to each taxon. For MAG classification, it reports the number of MAGs per taxon.

### Generation of contigs for clade exclusion benchmarking datasets

To test the performance of the algorithm in a first benchmark, we artificially generated contigs from known genome sequences in the RefSeq database [35] (Additional file 1: Table S6). We randomly downloaded one genome per taxonomic order from bacterial RefSeq on July 7, 2017 (163 orders in total) and cut the genomes into at most 65 non-overlapping contigs, generating a set of ~ 10,500 contigs with known taxonomic affiliation. Contig lengths were based on the length distribution of eight assembled real metagenomes deposited in the Sequence Read Archive (SRA) [36] (assembly with metaSPAdes v3.10.1 [4] after quality filtering with BBDuk that is included with BBTools v36.64 (https://sourceforge.net/projects/bbmap/), see Additional file 1: Table S6), with a minimum length of 300 nucleotides. This was done ten times to construct ten different benchmarking datasets sampled from 163 different genomes, each from a different taxonomic order.

Viruses remain vastly under-sampled, and the sequences in the database remain a small fraction of the total viral sequence space [37]. Moreover, the hierarchy of the viral taxonomy is not as deeply structured as the taxonomy of cellular organisms [38]. Based on these considerations, we did not explicitly assess the performance of our tool on viral sequences. However, we expect that classification of viruses will be readily possible when closely related viruses are present in the reference database.

### Reference databases with increasing levels of unknownness

The benchmarking datasets generated above are derived from genomes whose sequences are also present in the reference database, corresponding to the perhaps unlikely scenario where the query sequences in the metagenome are identical to known strains in the database. To benchmark our tools in the context of discovering sequences from novel taxa, we next generated novel reference databases with increasing levels of unknownness by removing specific taxonomic groups from nr. In addition to the original nr database (known strains), three derived databases were constructed to reflect the situation of discovering novel species, genera, and families. This was done by removing all proteins that are only present in the same species, genus, or family as any of the 163 genomes in the benchmarking dataset. To do this, either we removed the sequences from the database itself, or if a protein was identical in sequence to a protein in another clade, we changed the protein accession to taxonomy id mapping file to exclude the query taxon. In contrast to many other taxonomic classification tools, all the programs that we compared (CAT, DIAMOND best-hit, LAST+MEGAN-LR, and Kaiju) allowed such custom files to be used. The three reduced databases and associated mapping files thus reflect what nr would have looked like if the species, genus, or family of the genomes present in the benchmarking dataset were never seen before. This was done independently for each of the ten different benchmarking datasets, resulting in a total of 30 new reference databases to rigorously test the performance of our sequence classification tools in the face of uncharted microbial sequences. Simulating unknownness like this provides a better benchmark for classification of unknown sequences than a leave-one-out approach where only the query genome is removed from the reference database(e.g., [13, 17]), because close relatives of the query may still be present in the latter case.

### Programs, parameters, and dependencies

Nr database and taxonomy files were downloaded on November 23, 2017. Prodigal v2.6.3 [32] was used to identify ORFs on the simulated contigs. DIAMOND v0.9.14 [25] was used to align the encoded proteins to the reference databases for CAT and for the DIAMOND best-hit approach. Kaiju v1.6.2 [13] was run both in MEM and Greedy mode with SEG low complexity filter enabled. The number of mismatches allowed in Greedy mode was set to 5. For LAST+MEGAN-LR, LAST v914 [18] was used to map sequences to the databases with a score penalty of 15 for frameshifts, as suggested in [17]. Scripts in the MEGAN v6.11.7 [17] tools directory were used to convert LAST output to a classification file. The maf2daa tool was used to convert LAST output to a .daa alignment file. The daa2rma tool was used to apply the long-read algorithm. “--minSupportPercent” was set to 0 and the LCA algorithm to longReads, and the longReads filter was applied. “--topPercent” was set to 10 and “--lcaCoveragePercent” to 80 (MEGAN-LR defaults). The rma2info tool was used to convert the generated .rma file to a classification file. When a reduced database was queried, the appropriate protein accession to taxonomy id mapping file was supplied via its respective setting (see the section “Reference databases with increasing levels of unknownness” above).

### Scoring of contig classification performance

For contig classification, we scored (i) the fraction of classified contigs, (ii) sensitivity, (iii) precision, and (iv) mean and median rank of classification (Additional file 2: Figure S8). Classifications were compared at the taxonomic ranks of species, genus, family, order, class, phylum, and superkingdom. In those cases where *f* < 0.5 and multiple classifications reached the mbs threshold, we chose the lowest classification that reached a majority vote (i.e., as if *f* = 0.5) for calculating the four performance measures i–iv. This means CAT classifications were more conservative in those (rare) cases. Contigs with a classification higher than the superkingdom rank (e.g., “cellular organisms” or “root”) were considered unclassified, as these classifications are trivially informative in our benchmark. For all tools, a classification was considered correct if it was a subset of the true taxonomic lineage, regardless of rank of classification. If a classification was consistent with the true taxonomic lineage but classified too specifically (e.g., at the species rank whereas the query is a novel family), it was considered incorrect. For classifications that are shown per rank, only that part of the lineage that is too specific is considered incorrect.

The mean and median taxonomic rank of classification were calculated for all classified contigs, where the ranks species-phylum were given the integer values 0–6, respectively. Even though the true distance between taxonomic ranks may vary [39], calculating mean taxonomic rank in this fashion does serve as a proxy to show that classifications are called at higher taxonomic ranks “on average” under certain parameter conditions or, e.g., with higher divergence of the query sequence from the reference database. Sensitivity and precision were scored as (correctly classified/total number of contigs) and (correctly classified/total number of classified contigs), respectively. Thus, all performance measures are a property of the whole contig set and not of single taxonomic classifications as with some measures in the CAMI challenge benchmark further on. Wherever error bars are shown, they represent the maximum and minimum values out of the ten benchmark datasets.

### CAMI high-complexity gold standard benchmark

In a second benchmark, we downloaded the high-complexity gold standard assembly together with the taxonomy files and NCBI RefSeq database (dated January 30, 2015) that was supplied with the CAMI challenge [22]**.** We ran CAT on the assembly with RefSeq and nr (dated January 23, 2015) as reference databases. Importantly, both databases did not contain any of the query sequences yet.

We scored performance in exactly the same way as in the CAMI challenge, which allows us to compare the results of CAT to any of the taxonomic classifiers tested (“taxonomic binners”). In short, all four measures (accuracy, misclassification, average precision, average recall) are a function of the number of classified base pairs and not of classified contigs as in the benchmark above. If a tool classifies a sequence on a taxonomic rank that is not present in the gold standard, it is not taken into account. Thus, there is no penalty for classifications that are too specific. Accuracy is (number of correctly classified base pairs/total number of base pairs), misclassification (number of incorrectly classified base pairs/total number of base pairs), and both are thus a property of the whole assembly. Precision is a measure of the purity of a predicted taxonomic bin (i.e., all sequences from a single predicted taxon) with (number of correctly assigned base pairs/total assigned base pairs). Average precision is the mean precision of all predicted taxonomic bins and is thus very sensitive to misclassified small bins. Therefore in [22] in addition to precision measures of the full data, small bins summing up to 1% of the data are excluded and precision is recalculated. We did the same. Recall is a measure of the completeness of a real taxon bin (i.e., all sequences from a single query taxon), with (number of correctly assigned base pairs/real number of base pairs). Average recall is mean recall for all real taxon bins.

For a comparison with all taxonomic classifiers tested in the CAMI challenge, we downloaded the summaries from https://github.com/CAMI-challenge/firstchallenge_evaluation/tree/master/binning/tables/plot/supervised/summary_high.csv and https://github.com/CAMI-challenge/firstchallenge_evaluation/tree/master/binning/tables/plot/supervised/summary99_high.csv.

### MAG classification

For a third benchmark, 913 high-quality draft genome bins (MAGs) (completeness ≥ 80%, contamination ≤ 10%) from the cow rumen generated with both conventional metagenomics as well as Hi-C binning methods [7] were downloaded from the DataShare of the University of Edinburgh (https://datashare.is.ed.ac.uk/handle/10283/3009). Taxonomic classification of the MAGs was downloaded from the supplementary data that accompanies the paper and manually corrected if the names did not match our taxonomy files (Additional file 1: Table S5). To save disk space on the alignment file being generated, we ran BAT on batches of 25 genomes each. Akin to the contig classification case in the first benchmark, we only considered classifications by BAT at official taxonomic ranks and chose the majority classification in those cases were BAT gave more than one classification for a MAG (i.e., as if *f =* 0.5 for that MAG) resulting in more conservative classifications.

To manually assess the 28 MAGs whose classification was inconsistent with the published classifications, we created a phylogenomic tree of those bins together with closely related genomes that were downloaded from PATRIC [40] on January 16, 2018. CheckM v1.0.7 [20] was used to extract 43 phylogenetically informative marker genes that were realigned with ClustalOmega v1.2.3 [41]. We concatenated the alignments to create a superalignment and included gaps if a protein was absent. We constructed a maximum likelihood tree with IQ-TREE v1.6.3 [42], with ModelFinder [43] set to fit nuclear models (best-fit model LG+R7 based on Bayesian Information Criterion), including 1000 ultrafast bootstraps [44]. Per clade, rooted subtrees were visualized in iTOL [45].

We classified the MAGs with 2 MAG classification tools that are still under development, lastTaxa (https://gitlab.com/jfroula/lasttaxa) and GTDB-Tk v0.2.2 (https://github.com/Ecogenomics/GTDBTk). LastTaxa predicts ORFs with Prodigal and searches the nr database with LAST, after which classification is based on the majority classification of individual ORFs. LastTaxa was run on the same nr dataset as BAT, and they can thus be directly compared. GTDB-Tk first identifies marker genes and places the MAG in a reference genome tree based on these marker genes (see also [39]). GTDB-Tk was run with the classify workflow with release 86 of the GTDB-tk reference database. This database was constructed after the publication of [7]. The results of these comparisons can be found in Additional file 1: Table S5.

### Usage of computer resources

Run time and peak memory usage were estimated with the Linux/usr/bin/time utility. Elapsed wall clock time and maximum resident set size were scored for runs of CAT, MEGAN-LR, and Kaiju, classifying contig set #1 (10,533 contigs, see Additional file 1: Table S6) with the nr reference database. All tools were run with default parameter settings. Runs were performed on a machine with an Intel Xeon Gold 6136 Processor, 128 GB of memory, 24 cores, and 48 threads. Whenever one of the programs allowed for the deployment of multiple threads, all were used.

We estimated run time and peak memory usage for CAT, MEGAN-LR, Kaiju, and recent versions of the CAMI tools on the CAMI high-complexity dataset, with the NCBI RefSeq database that was supplied with the CAMI challenge as a reference. PhyloPythiaS+ was excluded because it needs a custom database that cannot be constructed based on RefSeq. The CAMI tools were run as suggested in their respective manuals and/or as done in the CAMI challenge (see Additional file 1: Table S4). MEGAN was run on a single metagenomic read file (out of 5 in the challenge); all the other tools were run on the gold standard assembly (42,038 contigs). Runs were performed on a machine with an Intel Xeon E5-2667 v3 Processor, 512 GB of memory, and 16 cores/threads. Whenever one of the programs allowed for the deployment of multiple threads, all were used.

CAT and BAT have been tried and tested on 128 GB machines.

## Supplementary information

**Additional file 1: Tables S1-S6.** Supplementary tables. (XLSX 7 mb)

**Additional file 2: Figures S1-S8.** Supplementary figures. (PDF 1.55 mb)

**Additional file 3.** Review history. (DOCX 21.7 kb)

## Data Availability

CAT and BAT are available under the MIT License at GitHub [46]. A version of the source code used in this manuscript is deposited on Zenodo [47]. All benchmarking datasets and reference databases with increasing levels of unknownness are available from the authors upon request. The contigs in the first benchmark are based on Bacterial RefSeq [35] and the databases based on nr [33]. The second benchmark is the CAMI benchmark [22], and the third is from Stewart et al. [7].
