# Supplementary material for: Robust taxonomic classification of uncharted microbial sequences and bins with CAT and BAT
Source: Genome Biol. 2019 Oct 22;20:217. doi: 10.1186/s13059-019-1817-x (PMC6805573; doi:10.1186/s13059-019-1817-x)
Supplement: Supplementary file 2 — Additional file 2: Figures S1-S8. Supplementary figures. (PDF 1.55 mb) [file 13059_2019_1817_MOESM2_ESM.pdf]

Fig. S1

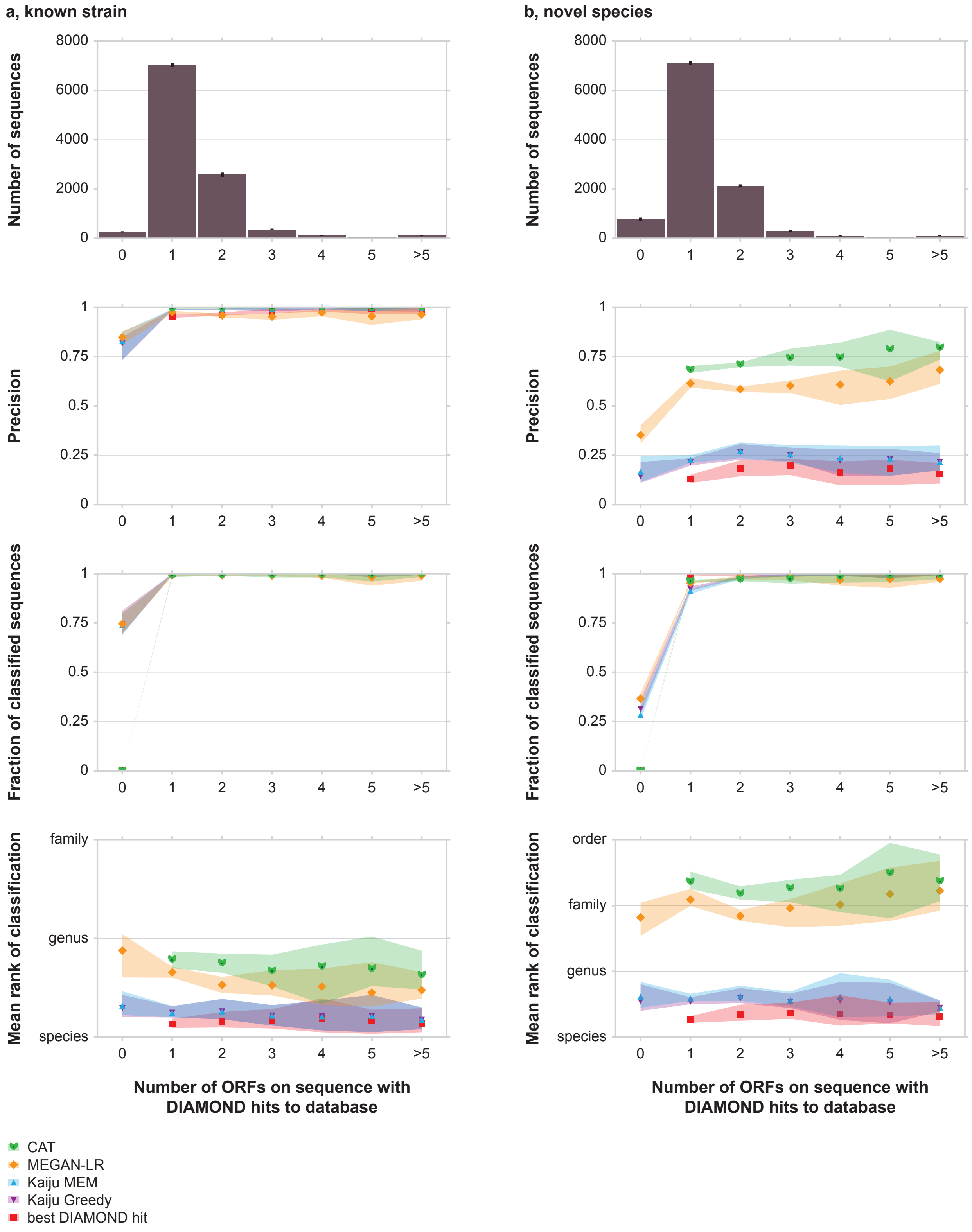
a, known strain
b, novel species
Number of sequences
8000
6000
4000
2000
0
0
1
2
3
4
5
>5
Precision
1
0.75
0.5
0.25
Fraction of classified sequences
Mean rank of classification
family
genus
species
order
Number of ORFs on sequence with DIAMOND hits to database
CAT
MEGAN-LR
Kaiju MEM
Kaiju Greedy
best DIAMOND hit

Fig. S1 - continued

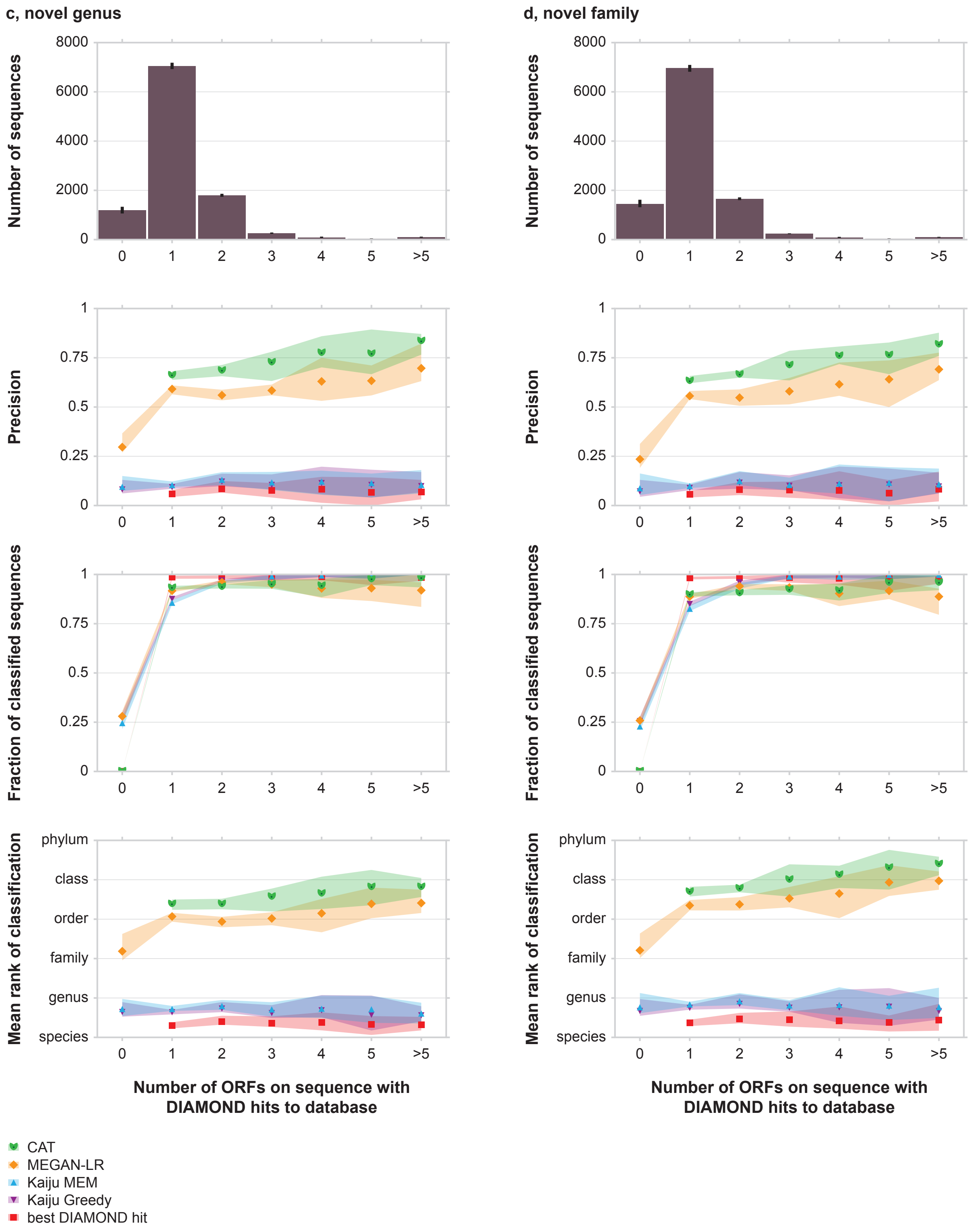

Fig. S1. Classification performance of CAT, LAST+MEGAN-LR, Kaiju, and DIAMOND best-hit with sequences binned according to the number of ORFs on the sequence with DIAMOND hits to the database. (**a**) Classification of known sequences, (**b-d**) classification of simulated novel taxa for different levels of divergence from reference databases. Sequences can fall in the 0 bin for three reasons: no ORFs are recognised on the sequence, ORFs are predicted but they do not have any hits to the database, or the ORF does have hits but its accession number cannot be found in the NCBI taxonomy files. In those cases where 1 or more ORFs on a sequence have DIAMOND hits, the fraction of classified sequences by CAT and DIAMOND best-hit can only be lower than 1 when some classifications are made at trivially informative taxonomic ranks such as ‘cellular organisms’ or ‘root’. Black error bars (in top figures) and shaded areas indicate maximum and minimum values out of ten benchmarking datasets.

Fig. S2

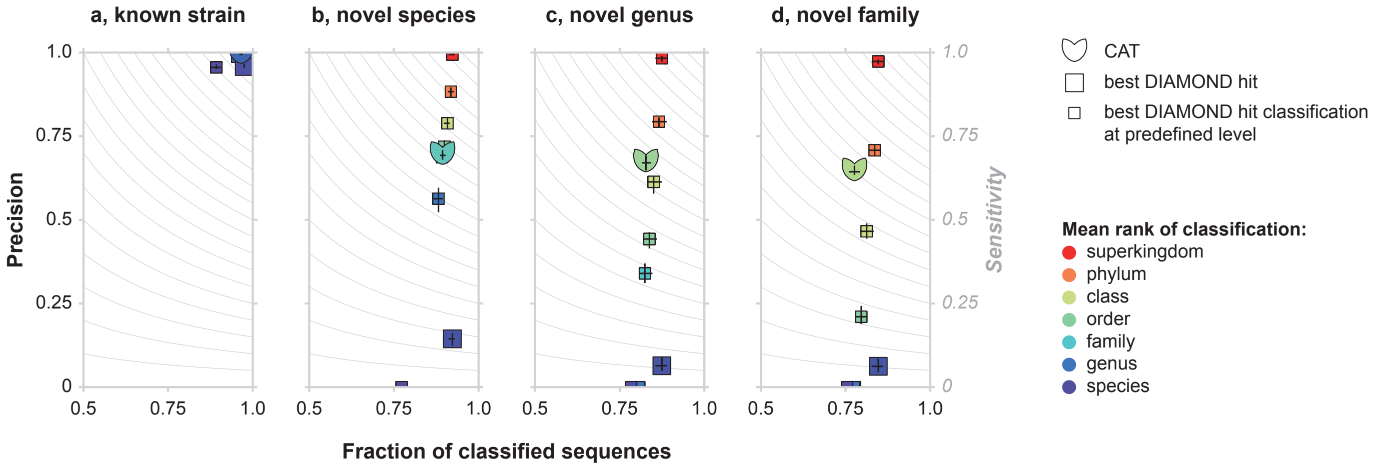

Fig. S2. Classification performance of CAT, DIAMOND best-hit, and DIAMOND best-hit with different taxonomic rank cut-offs. (**a**) Classification of known sequences, (**b-d**) classification of simulated novel taxa for different levels of divergence from reference databases. Black bars indicate maximum and minimum values out of ten benchmarking datasets, bars cross at the means. Colour coding indicates the mean taxonomic rank of classification averaged across the then benchmarking datasets (minimum and maximum values not shown for brevity).

Fig. S3

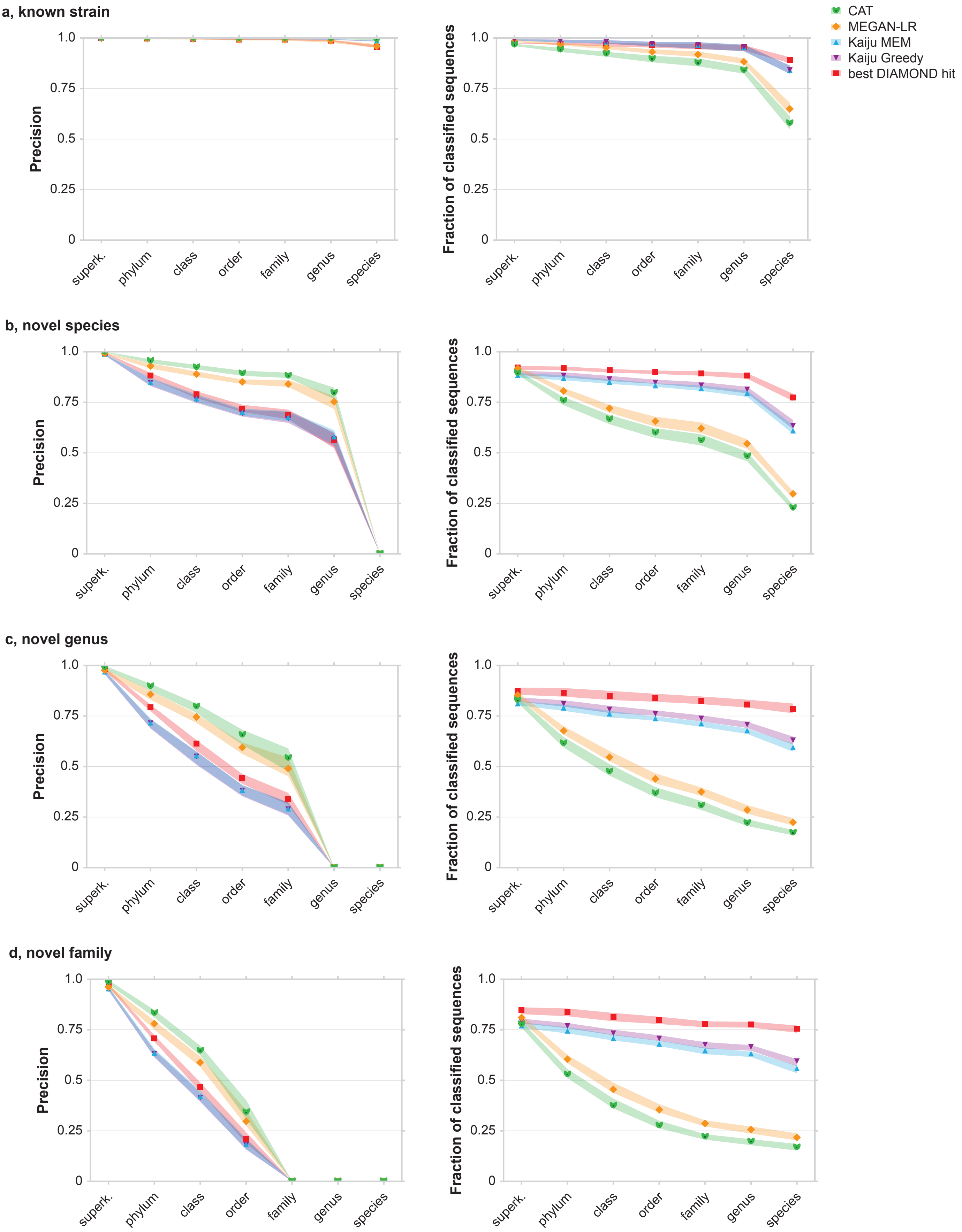

Fig. S3. Classification performance of CAT, LAST+MEGAN-LR, Kaiju, and DIAMOND best-hit for different levels of unknownness across taxonomic ranks. (**a**) Classification of known sequences, (**b-d**) classification of simulated novel taxa for different levels of divergence from reference databases. Shaded areas show maximum and minimum values across the ten benchmarking datasets.

Fig. S4

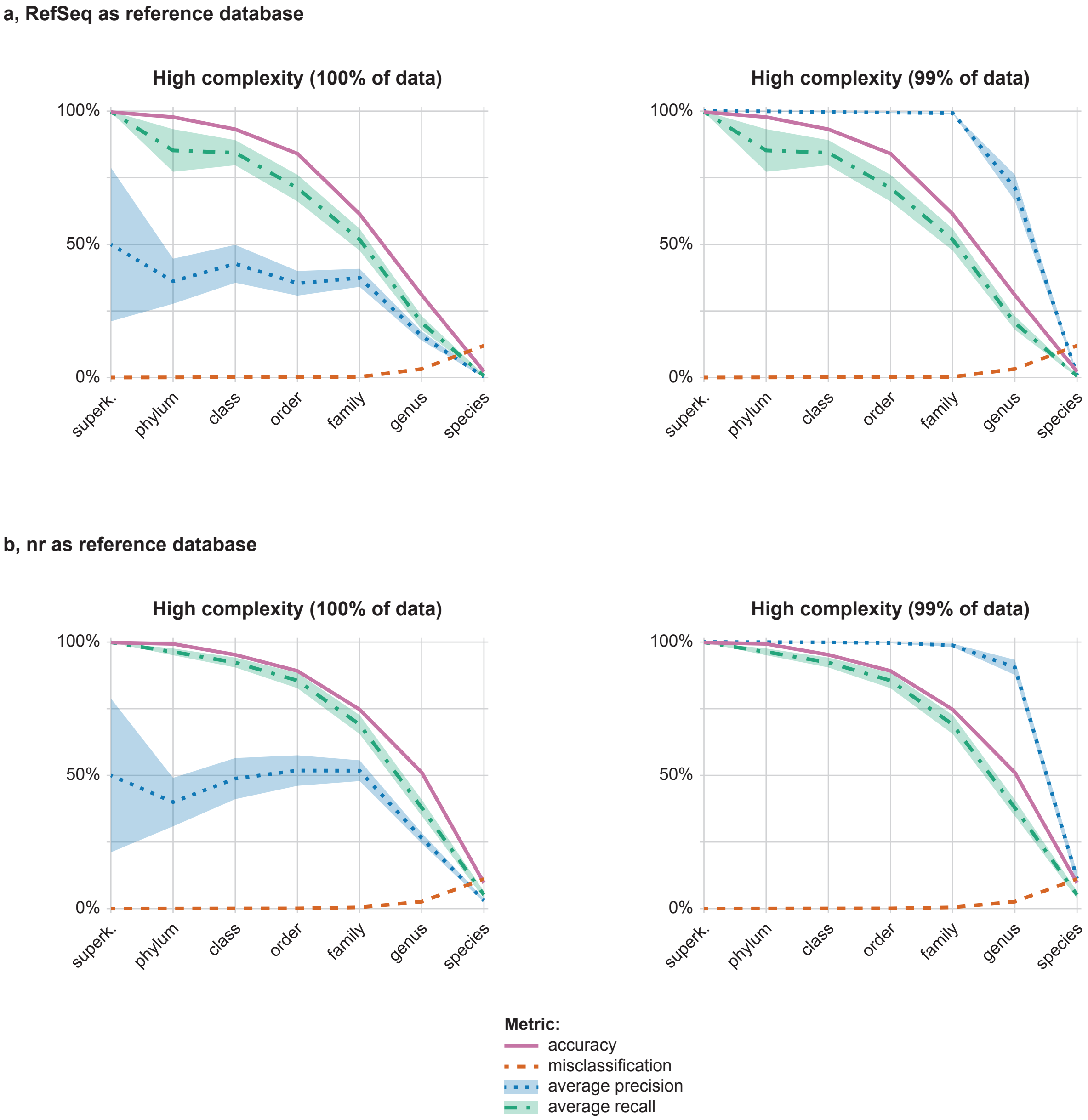

Fig. S4. Classification performance of CAT on the CAMI high-complexity gold standard assembly with (**a**) RefSeq as reference database, and (**b**) nr as a reference database. Plots on the left show average precision if all taxonomic annotations are included, plots on the right if shortest classifications representing less than 1% of the total assembly length are excluded. Accuracy, misclassification, and average recall are the same in both plots. Note that measures are the same as those calculated in the CAMI challenge, and thus precision here reflects something different from the precision we used earlier in the clade exclusion experiments. Shaded areas show the Standard Error of the Mean (SEM) for average precision and average recall. a can be compared to Supplementary Figure 18 and b to Supplementary Figure 19 in the CAMI paper (Sczyrba et al., 2017).

Fig. S5

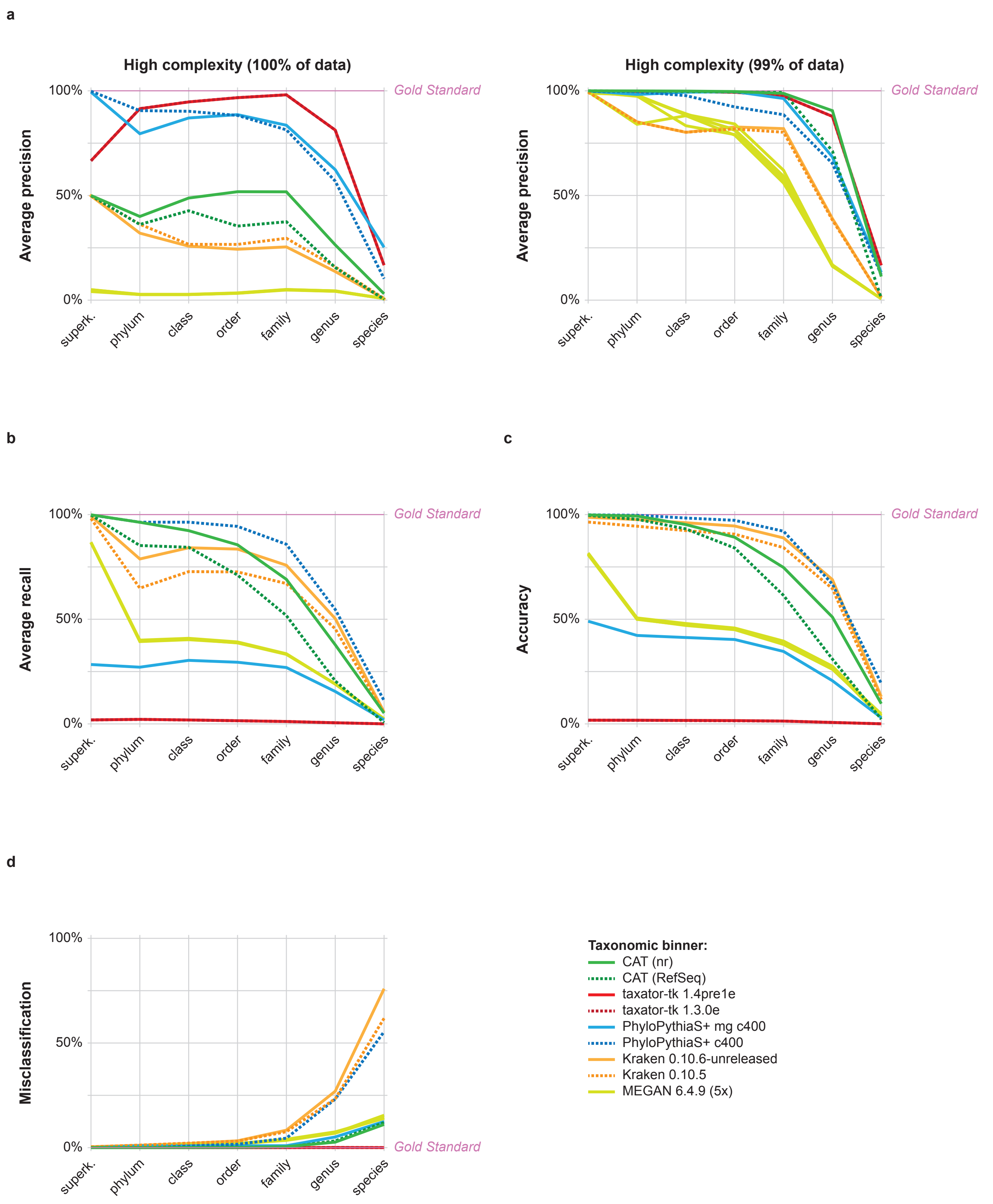

Fig. S5. Comparison of CAT with RefSeq and nr as reference database against the taxonomic classifiers tested in the CAMI challenge, for (**a**) average precision, (**b**) average recall, (**c**) accuracy, and (**d**) misclassification. The left plot in a shows average precision if all taxonomic annotations are included, the right plot if shortest classifications representing less than 1% of the total assembly length are excluded.

Fig. S6

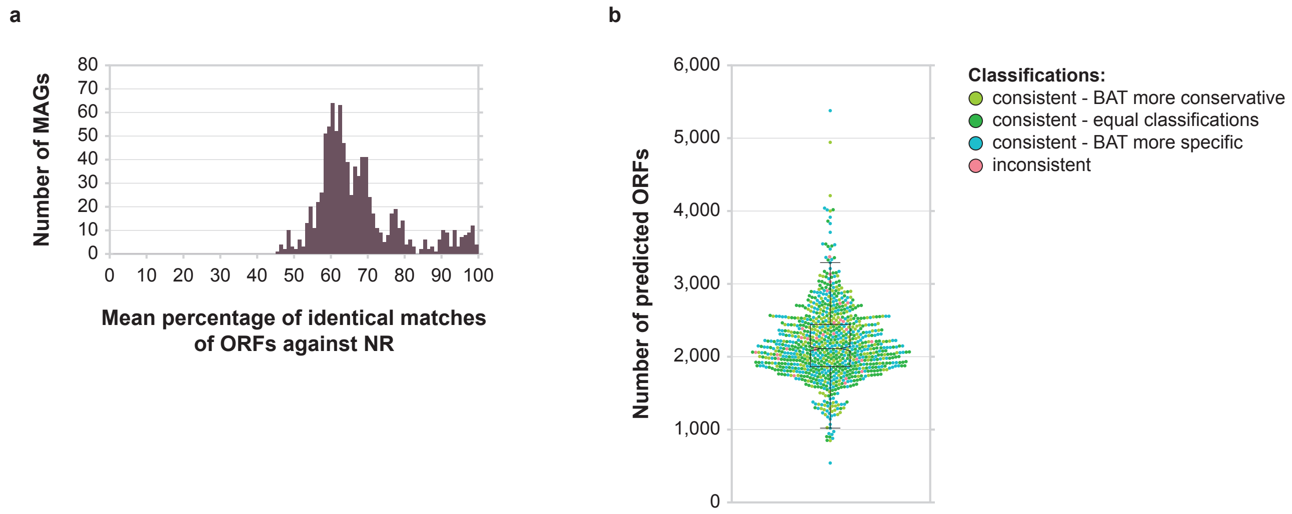

Fig. S6. Predicted ORFs on 913 MAGs. (**a**) Average percentage of identical matches with the best DIAMOND hit in the nr database for all predicted ORFs in a MAG. The wide distribution shows that the MAGs represent a wide range of novelty, i.e. most MAGs are organisms that are not present in the nr database yet. (**b**) Swarmplot showing the number of predicted ORFs per MAG. MAGs are coloured as in Fig. 6 (r = 5, f = 0.3).

Fig. S7

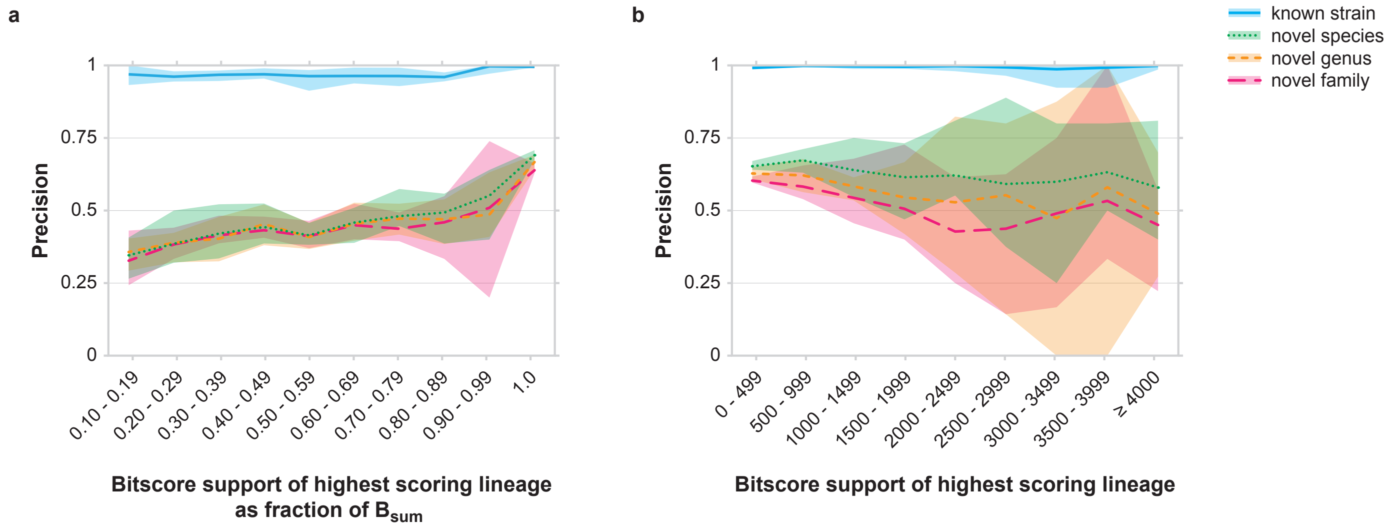

Fig. S7. Classification performance of CAT binned per (**a**) fraction of summed bit-score support and (**b**) total bit-score support. CAT was run with *f* = 0.1 and only the values of the lowest classification of the chosen lineage are shown. If multiple lineages have a score higher than 0.1, the majority classification is chosen (i.e. the sequence is classified as if *f* = 0.5). Shaded areas indicate maximum and minimum values out of ten benchmarking datasets. Since only values of the lowest classification are shown per lineage, and taxon classifications higher up the lineage have a higher chance of being correct and also higher support values, the relation between fraction of summed bit-score support and precision is even more pronounced for all taxa in a lineage.

Fig. S8

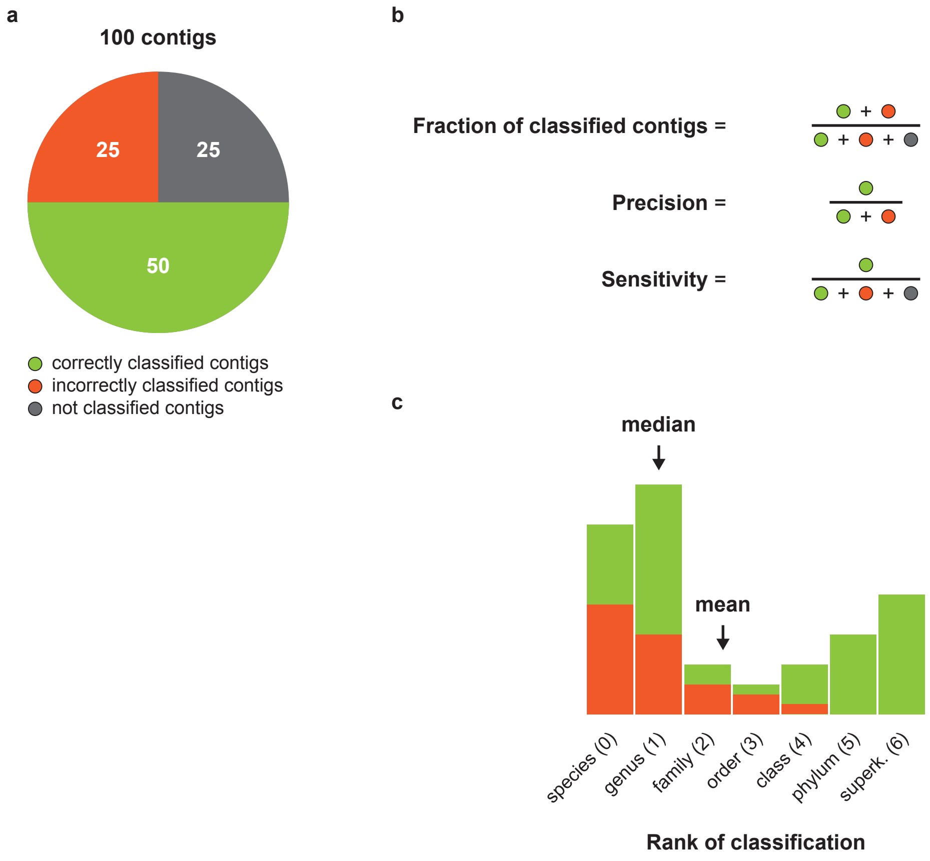

Fig. S8. Measuring performance for contig classification. (**a**) Example contig set. Classifications above superkingdom rank (e.g. 'cellular organisms' or 'root') are considered not classified. Half of the total classifications is contained within the true taxonomic lineage and is thus scored as correct, and a quarter is not. If a classification is in the correct lineage but too specific, it is considered incorrect. (**b**) Measures of performance. Precision is a measure for how trustworthy a classification is, sensitivity for how much of the total data is correctly classified. Sensitivity is fraction of classified contigs *x* precision. (**c**) Mean and median taxonomic rank of classification are calculated for all classified contigs (75 in the example), where the ranks species-phylum are given the integer values 0-6, respectively, allowing a mean to be calculated.
