# Supplementary material for: Robust taxonomic classification of uncharted microbial sequences and bins with CAT and BAT
Source: Genome Biol. 2019 Oct 22;20:217. doi: 10.1186/s13059-019-1817-x (PMC6805573; doi:10.1186/s13059-019-1817-x)
Supplement: Supplementary file 3 — Additional file 3. Review history. (DOCX 21.7 kb) [file 13059_2019_1817_MOESM3_ESM.docx]

Review History

**First round of review**

**Reviewer 1**

**Were you able to assess all statistics in the manuscript, including the appropriateness of statistical tests used? No**

**Were you able to directly test the methods? No**

**Comments to author:**

Classifying contigs and genome bins assembled from metagenomic datasets is a challenging process, and the current methods (homology-based or phylogeny-based) are either not accurate or not automated. The annotation becomes more unreliable for sequences derived from relatively unknown taxa. In this work the authors developed an automated tool suite to classify the taxonomy of metagenomic contigs and bins, by parsing and integrating homology search results from multiple predicted ORFs. They evaluated the accuracy of their software tools on both synthetic and real-world datasets, and showed superior performance over alternative software. The majority of the manuscript was written in clear language and easy to follow.

General comments:

1. How well the bit score correlates with classification accuracy? Does it translate to classification confidence like BLAST bit scores?

2. As the classification happens at every rank, in theory it may produce inconsistencies, for example, a bin's known Family classification inferred from its Genus conflicts its predicted Family. In practice this actually happens, especially when we deal with species from novel ranks.  The author may need to consider how to resolve these inconsistencies.

3. There should be some discussions about the robustness on annotating very novel species, which is quite common in metagenome datasets. For example, what if the majority of the proteins in a bin/contig have no hits? Why the simulation stopped at novel Family, does the author suggest for novel Order, Class, readers should resort to phylogeny-based approaches?

Specific comments:

L124-129. It is a bit confusing here. I guess the authors meant increasing r causes less contigs classified, more contigs classified at higher rank, while decreasing r causes the opposite effect?

L145-148. Kaiju or Diamond are not designed to annotate long contigs with multiple ORFs. For a fair comparison I would suggest comparing CAT to GTDB-tk (https://github.com/Ecogenomics/GTDBTk) and LastTaxa (https://gitlab.com/jfroula/lasttaxa), both tools seem to use a similar strategy for annotation.

**Reviewer 2**

**Were you able to assess all statistics in the manuscript, including the appropriateness of statistical tests used? Yes. I have a comment about the statistics.**

**Were you able to directly test the methods? No**

**Comments to author:**

The manuscript presents two taxonomic classifiers, Contig Annotation Tool (CAT) and Bin Annotation Tool (BAT), both of which integrate multiple open reading frames as signals to separately classify long sequences and metagenome-assembled genomes. I agree with the author's observations that many existing taxonomic classifiers generated too specific, but incorrect, classifications. Their new algorithms were shown to provide significant improvement to address this challenge. These two algorithms will be broadly useful for metagenomics studies.

Major comments:

1. The algorithm used a heuristic approach based on a set of arbitrary parameters, including r, f, and mbs, for making the classification. The authors did perform parameter optimization to set good default values for these parameters. However, the results don't have rigorously-defined statistical meaning. It would be better if the algorithm can provide uncertainty quantification for its classification output based on some statistical models. For example, in Figure 1c, can the algorithm calculates this contig to have 60% chance to be E. coli, 80% chance to be in the Escherichia genus, etc? In this way, the users can use this statistical confidence measure to adjust the level of taxonomic classification precision at a given statistical confidence.

2. I expect that the performance of this algorithm will increase with the number of ORFs in a config or a MAG. In many metagenome assemblies, a large fraction of contigs are short contigs with very few genes. The authors should benchmark the performance of different algorithms for various sizes of contigs.

3. The authors directly compared their new algorithms with LAST+MEGAN-LR, Kaiju, and a conventional best-hit approach with DIAMOND, which are quite limited. They used the reported results of taxator-tk, PhyloPhythiaS and MEGAN from CAMI in 2015. I think it is more consistent to benchmark the latest versions of these algorithms with benchmark 1 or benchmark 2 under identical settings. This will provide a comparison of the computational cost of all these algorithms (Figure 4)

**Authors Response**

**Point-by-point responses to the reviewers’ comments:**

1.1) How well the bit score correlates with classification accuracy? Does it translate to classification confidence like BLAST bit scores?

Answer: To address this question, we performed a new analysis that is now presented in Supplementary Figure 7 and accompanying text, that explores the classification precision for (i) the fraction of total bit-score support (Supplementary Figure 7A) and (ii) the uncorrected bit-score values (Supplementary Figure 7B). As expected, we observed that a higher fraction of summed bit-score support also shows on average higher precision. As explained in the text, CAT uses this summed bit-score statistic to assess the final taxonomic classification. Notably, the uncorrected bit-score values do not correlate with precision (Supplementary Figure 7B). The fraction of summed bit-score support is written to CAT and BAT output files and can thus be used to judge the classification at different taxonomic ranks. From a user perspective, it is also important to note that precision increases with a higher number of classified ORFs on a contig (newly added Supplementary Figure 1 , see points 1.3 and 2.2 below). This information is also written to the output file.

1.2) As the classification happens at every rank, in theory it may produce inconsistencies, for example, a bin's known Family classification inferred from its Genus conflicts its predicted Family. In practice this actually happens, especially when we deal with species from novel ranks.  The author may need to consider how to resolve these inconsistencies.

Answer: With the default cut-off parameter value for CAT classification (*f* = 0.5 or higher) inconsistencies cannot happen. This is now clarified in the text. Only if *f* < 0.5 then multiple classifications are possible. In those cases, CAT and BAT write those multiple classifications to the output file with support values for each. Users can decide which (rank of) classification to choose based on the bit-score support values (see also point 1.1 above). We have added a paragraph in the Methods section to clarify this. Note that for the analyses in our manuscript we chose *f* = 0.5 in case of multiple classifications (the majority of the bit-score support for a contig’s classification), and we suggest that this is a safe bet for large scale studies.

1.3) There should be some discussions about the robustness on annotating very novel species, which is quite common in metagenome datasets. For example, what if the majority of the proteins in a bin/contig have no hits?

Answer: We have tried to remove all ambiguity as to what constitutes “very novel species”. Our first benchmark simulates novel species, novel genera, and novel families, showing that they can be very robustly classified (see also point 1.4 below). We have added Supplementary Figure 1 that shows the distribution of the number of ORFs with a DIAMOND hit on a sequence in the top panel. This new figure shows that sequences with a higher number of ORFs with homologs in the database have on average a higher precision of classification. We now discuss this analysis in the manuscript, together with the implication in the context of the common novel species (or genera, families) in metagenomic datasets.

1.4) Why the simulation stopped at novel Family, does the author suggest for novel Order, Class, readers should resort to phylogeny-based approaches?

Answer: we pragmatically stopped the simulation in our first benchmark at novel family because we picked one genome per order to avoid taxonomically biasing our benchmarking experiment. There are enough orders in our RefSeq dataset for a meaningful benchmark (163), but only 74 classes. Importantly however, CAT and BAT also perform very well when classifying novel orders, classes, or phyla. This is shown in the third benchmark (MAG classification), where there is a range of novelty on all taxonomic levels ranging from species to super kingdom. We have added a sentence in the introduction to clarify this.

1.5) It is a bit confusing here. I guess the authors meant increasing r causes less contigs classified, more contigs classified at higher rank, while decreasing r causes the opposite effect?

Answer: We agree that the wording was a bit unclear, we have rephrased this. The reviewer is correct in their interpretation.

1.6) Kaiju or Diamond are not designed to annotate long contigs with multiple ORFs. For a fair comparison I would suggest comparing CAT to GTDB-tk (https://github.com/Ecogenomics/GTDBTk) and LastTaxa (https://gitlab.com/jfroula/lasttaxa), both tools seem to use a similar strategy for annotation.

Answer: We are happy to see more tools being developed for the classification of MAGs for reasons we outline in the manuscript. Since both of the tools mentioned by the reviewer are not published yet, we did not originally include them in our benchmarks. However, as part of our third benchmark (MAGs classification) we have now added a new table (Supplementary Table 5) that compares the classifications of BAT, lastTaxa, and GTDB-Tk, and discuss these results in the text. In absence of a publication, we cite the respective GitHub/GitLab repositories for these tools.

2.1) The algorithm used a heuristic approach based on a set of arbitrary parameters, including r, f, and mbs, for making the classification. The authors did perform parameter optimization to set good default values for these parameters. However, the results don't have rigorously-defined statistical meaning. It would be better if the algorithm can provide uncertainty quantification for its classification output based on some statistical models. For example, in Figure 1c, can the algorithm calculates this contig to have 60% chance to be E. coli, 80% chance to be in the Escherichia genus, etc? In this way, the users can use this statistical confidence measure to adjust the level of taxonomic classification precision at a given statistical confidence.

Answer: The reviewer is right that the CAT/BAT classification scores are not true statistical measures such a blast e-value, see also point 1.1 above. (Note that this is also the case for many other popular computational and experimental tools.) Since the bit-score support is calculated for every rank, a user can decide per rank whether to trust its classification; i.e. a sequence may be classified with 60% bit-score evidence to *Escherichia coli* and with 80% bit-score evidence to *Escherichia*. We have now expanded our discussion in the manuscript on the bit-score support and its interpretation, stating clearly that it is not a true statistical measure and showing its effect on prediction performance in the newly added Supplementary Figure 7. Based on the data presented in these figures, we considered processing the bit-score to fit an uncertainty quantification, but decided against it as this score would invoke an arguably intractable formula to calculate one from the other. We concluded that this would obscure the actual evidence for a certain classification (the bit-score) and be confusing rather than helpful to the user.

2.2) I expect that the performance of this algorithm will increase with the number of ORFs in a config or a MAG. In many metagenome assemblies, a large fraction of contigs are short contigs with very few genes. The authors should benchmark the performance of different algorithms for various sizes of contigs.

Answer: We thank the reviewer for this useful suggestion that is related to point 1.3 above. The newly added Supplementary Figure 1 shows that the reviewer's hypothesis is correct; more ORFs on a sequence means a higher precision. We have discussed this in the text as well.

2.3) The authors directly compared their new algorithms with LAST+MEGAN-LR, Kaiju, and a conventional best-hit approach with DIAMOND, which are quite limited. They used the reported results of taxator-tk, PhyloPhythiaS and MEGAN from CAMI in 2015. I think it is more consistent to benchmark the latest versions of these algorithms with benchmark 1 or benchmark 2 under identical settings. This will provide a comparison of the computational cost of all these algorithms (Figure 4).

Answer: In Figure 4 of our manuscript we only compared the first set of tools to CAT because they can be run on the same large reference protein database (nr), which is not the case for most of the CAMI tools. We agree that it would be useful to compare run time and RAM usage of these algorithms to the CAMI tools as well, which is why we have now added the new Supplementary Table 4. In this table, we compare run time and RAM usage of all the tools mentioned in the manuscript with RefSeq as a reference database, either on the DNA or protein level. (The only tool that we could not include is PhyloPhytiaS+ since it needs a custom database that cannot be built from RefSeq.) All tools were run with parameters as suggested in their respective manuals and/or as done during the CAMI challenge. We moreover show in Supplementary Table 4 how changing DIAMOND parameters can speed up run time for CAT.

**Second round of review**

**Reviewer 1**

My questions were sufficiently addressed during the revision. No further questions.

**Reviewer 2**

The authors addressed my comments to the best of their ability. The algorithm still lacks a solid statistical foundation, but fixing that would require development of a new algorithm, which is beyond the scope of this manuscript.
